# Folk medicine used to heal malaria in Calabria (southern Italy)

**DOI:** 10.1186/1746-4269-6-27

**Published:** 2010-09-18

**Authors:** Giuseppe Tagarelli, Antonio Tagarelli, Anna Piro

**Affiliations:** 1 Istituto di Scienze Neurologiche-CNR, Contrada Burga 87050 Mangone, Cosenza, Italy

## Abstract

In Italy, malaria was an endemic disease that was eradicated by the mid-20th century. This paper evaluates the prophylactic and therapeutic remedies used by folk medicine to cure malaria in Calabria (southern Italy).

The data has been collected by analysing works of physicians, ethnographers, folklorists and specialists of the study of Calabrian history between the end of the 19th century and the 20th century. The data collected have allowed us to describe the most common cures used by the Calabrian people to treat malaria and the most evident symptoms of this disease, such as intermittent fever, hepato-spleenomegaly, asthenia and dropsy. This approach uncovered a heterogeneous *corpus *of empirical, magical and religious remedies, which the authors have investigated as evidences of past "expert medicine" and to verify their real effectiveness in the treatment of malaria.

## Background

Malaria is an infectious disease that is caused by the *Plasmodium *parasite. This disease is transmitted to humans via the Anopheles mosquito. Malaria is a very ancient disease, and although it was not possible to prove its presence in ancient human bones, this disease was probably present among *Homo *genus ancestors [1]. Different populations, such as the Sumerians, Assyrian-Babylonians, Indians, Egyptians and Chinese, experienced seasonal and intermittent fevers [2]. In the Mediterranean area, particularly in Italy, malaria was an endemic disease that was eradicated by the mid-20th century. Moreover, the persistent and lasting presence of malaria determined an interesting state of debility of the affected subjects and a consequent weakening of the labour force, which led to some important and detrimental socio-economic consequences [3]. Folk medicine approaches were used in an attempt to treat several of the most evident effects of malaria, such as intermittent fever, hepato-spleenomegaly, asthenia and dropsy.

It is our aim in this work to identify folk medical cures that were used by the Calabrian people for the treatment of malaria, as evidenced in writings produced between the 19th and 20th centuries. The authors have also examined whether same remedies were already described by Pliny the Elder, Dioscorides, Galen and Serenus Sammonicus, so to be considered as evidence of past "expert medicine".

## Area of Study

**Figure 1:**
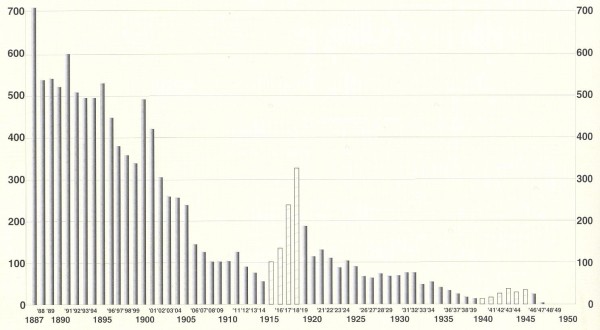
**Reduction in malaria mortality in Italy from 1887 to 1950 (number of deaths per 1,000,000 inhabitants)**. The white histograms refer to the years of the First and Second World War. Source: A. Coluzzi, modified by the authors.

**Figure 2:**
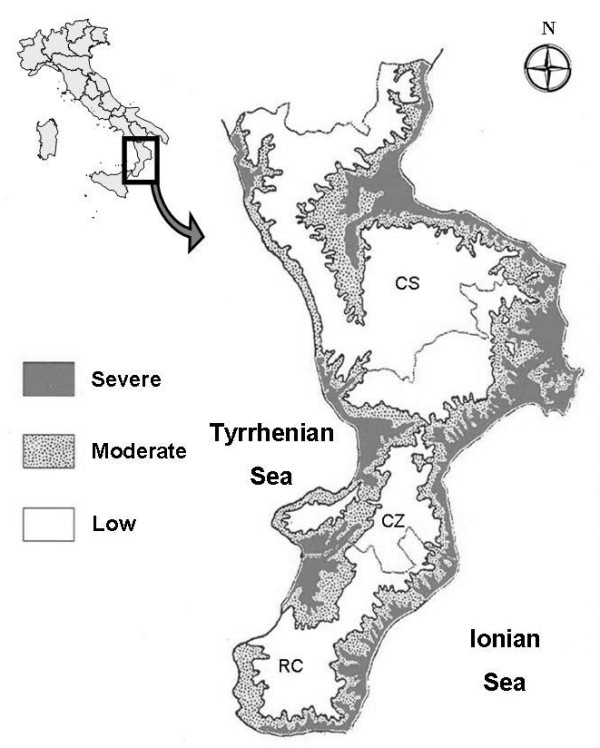
**Distribution of past malarial prevalence in Calabria (Southern Italy)**. The map shows the old administrative division in three provinces (CS Province of Cosenza, CZ = Province of Catanzaro; RC = Province of Reggio Calabria). Source: A. Tagarelli.

It is interesting to point out that in some peninsular and insular areas of Italy, despite all the drainage attempts initiated in the 16th century, malaria-associated mortality was only recently eradicated in the mid-20th century (Figure 1) [4]. Among the southern regions of Italy, Calabria was one of the regions that was most affected by malaria. The disease was endemic along its coasts (about 738 km), along its most important rivers (Mesima, Lao, Crati, Tacina and Neto) and within the valleys of its broad streams. The disease was prevalent in 52% of the Calabrian territory (7,877.31/15,080.32 km^2^) (Figure 2) [5]. Calabria showed both natural and antropic factors that favoured the spread of *Plasmodium*, as well as the endemic and century-old presence of malaria in its territory. Physical features that may have affected the spreading of malaria are represented by a rich hydrographic reticle and the occurrence of seismic phenomena (bradyseisms and earthquakes), which, at that time, contributed to increase the hydrogeological disorder, thus creating many different wet areas (for example, the single earthquake of 1783 created about 215 lakes), which are the favourite environment of the anopheles mosquito [6]. The antropic factors are represented fundamentally by latifundia, deforestation and the very poor social and economic conditions of the rural Calabrian people [7].

Calabria is the southernmost region of peninsular Italy; it borders with the Ionian Sea to the east and south, with the Tyrrhenian Sea to the west, and with the region Basilicata to the north, and it extends for about 250 km from north to south in the middle of the Mediterranean Sea. Calabria reaches 15,080 km^2 ^and 42% of its territory is represented by mountains: the Apennine mountain range - the southern Apennines, calcareous, with Pollino Massif (Serra Dolcedorme, 2267 m a.s.l.)- and the Calabrian Apennines, mainly siliceous - with the Coastal Range (M. Cocuzzo,1541 m a.s.l.), Sila Massif (M. Botte Donato,1929 m a.s.l.), Serre Calabre (M. Pecoraro, 1423 m a.s.l.) and Aspromonte Massif (Montalto, 1956 m a.s.l.)-. 49% of Calabria's territory is represented by hills and only 9% is flat. The plains are restricted to coastal areas and only three plains extend into the internal areas of the territory (Sibari plain, Saint'Eufemia plain and Gioia Tauro plain).

The climate is Mediterranean, with maximum precipitation during the winter and minimum in the summer and vice versa for the temperature. Precipitation is represented by about 1,041 mm of rainfall per year. The average temperature in the coldest month (January) is about 8.3°C and the warmest month (August) about 24.4°C, with an annual average of 15.8°C [8]. However strong meso-climatic variations occur depending on altitude, topographic features and location with respect to the sea.

From both an anthropological and an ethnobotanical point of view, Calabria is an interesting region, for the historical presence of several populations (Greeks, Romans, Byzantines, Arabs, Normans and Spanish) in the past that influenced the local culture [9]. Finally, it is interesting to note that the Arbëreshe community, of Albanian origins, settled in this region since the 16th century and is currently located in 25 communes in the provinces of Cosenza, Catanzaro and Crotone [10].

## Methods

This study is based on the analysis of works written by physicians, ethnographers, folklorists and specialists of the study of Calabrian history between the end of the 19th century and the 20th century, in particular when both ethnographic and anthropological research focused on the study of folk medicine. For this reason, the authors have also consulted the check-lists about works on calabrian folk medicine and beliefs, edited by Lombardi Satriani [11] and Cavalcanti [12]. The sources used in this work are listed in Table 1. The data collected has allowed us to describe the cures used by the Calabrian people to treat malaria and its most evident symptoms, such as intermittent fever, hepato-spleenomegaly, asthenia and dropsy. This approach uncovered a heterogeneous *corpus *of empirical, magical and religious remedies, which the authors have investigated as an "official medicine" to cure malaria in the treatises by Pliny the Elder (*Naturalis Historia*) [13-15], Dioscorides (*De Materia Medica*) [16], Galen (*Opera Omnia*) [17-21] and by Serenus Sammonicus (*Liber Medicinalis*) [22]. These authors, in fact, have influenced medical practice in latter centuries [23].

**Table 1:** Sources used for the research

Author	Period°	Description	Reference
Francesco Genovese	1912-1924	Physician, malariologist who wrote about malaria in Calabria.	[6,56,62]

Alessandro Adriano	1932	Physician whose daily experiences provided information about folk Calabrian medicine.	[65]

Giovanni De Giacomo	1892-1896	Folklorist who published many works about folk culture including therapeutic remedies to cure many diseases.	[26,54,55,57]

Gianbattista Marzano	1889-1928	Folklorist and historian who wrote about folk traditions in Laureana di Borrello (province of Reggio Calabria). He published a vocabulary of south Calabrian dialect with historical and folkloric notes as well.	[25,68]

Raffaele Lombardi Satriani	1916-1951	Folklorist and ethnographer who published many works about the Calabrian people.	[11,53]

Luigi Accattatis	1895	Historian and linguist who published a vocabulary of north Calabrian dialect with historical and folkloric notes.	[24]

Filippo Jacopo Pignatari	1894-1895	Man of letters who published many papers about Calabrian beliefs and traditions including the use of plants and animal to cure many diseases.	[58-60,67,75]

Luca De Samuele Cagnazzi	1811	Mathematician who edited the statistical report of the Kingdom of Naples of 1811.	[72]

Vincenzo Donnarumma	1951	Franciscan monk who wrote a book about the religious cult of the Madonna in the province of Cosenza.	[78]

Antonio Iannicelli	1991	Writer who published a book about Calabrian folk traditions.	[79]

Vincenzo Romeo	1946	Physician who published a work about anti-malaria prophylaxis.	[74]

Leopoldo Pagano	1853-1901	Historian and man of letters. He wrote an important book about the economy, history and environment of Calabria.	[66,70]

Placido Olindo Geraci	1957	Man of letters was author of a paper about folk Calabrian medicine.	[64]

Biagio Lanza	1853-1860	Physician and author of a paper about folk medicine in Cassano (province of Cosenza).	[63]

Raffaele Corso	1953	Along with Lombardi Satriani, he was one of the most important Italian folklorists and etnhographers. He wrote many works about amulets.	[77]

Vincenzo Brancia	1853-1860	Priest who wrote a paper about folk medicine in Nicotera (province of Catanzaro).	[69]

Lorenzo Galasso	1915	Priest who wrote a book about the Calabrian people	[76]

Michele Tenore	1827	Botanist who published a work on *Prunus spinosa *L.	[73]

Silvio Mollo	1930	Man of letters and author of a book about Calabrian folklore	[71]

° Refers to the year of publication of the works examined.

The plants used by Calabrian people to cure malaria (N = 53) have been identified as species because the sources described them with their scientific name (34% of cases, 18/53), or with their Italian name (60% of cases, 32/53). In the latter case, we attributed a plant to a species only if we were absolutely sure about it (for example "olivo" (olive) = *Olea europaea *L.); in the other cases we classified the plants only through their genus (for example "quercia" (oak) = *Quercus *spp.). Furthermore, due to the presence of different dialects in Northern, Central and Southern Calabria, the analysis of the different historical sources required the use of two historical dictionaries of Calabrian dialects [24,25], which were used to translate several ancient terms (that are not used today) into Italian in 4% of cases (2/53). This has allowed us to attribute the local name "vruca" to the genus *Tamarix *spp., while it has not allowed us (2% of cases, 1/53) to attribute the local name "catabuzzico" [26] to any genus. Finally, the plants used to cure malaria in Calabria (N = 52) have been compared with their pharmacological and biological properties [27-48] and with their current use in Calabrian folk medicine, defined by recent fieldworks [49-51] [Table 2].

**Table 2:** Medical use of plants to heal malaria in Calabria, bio-pharmacological properties and current use in Calabrian folk medicine.

Plants used to heal malaria in Calabria			Current use in Calabrian folk medicine°			Pharmacological/biological proprierties	Used part	References
			
Family/Scientific Name	Local Name	References	Aim	Used part	References			
Adoxaceae								

*Sambucus nigra *L.	savucu	[26,55]	Antirheumatic, arteriosclerosis, febrifugal, purgative, to treat swollen breast and legs, insect bites, toothache, colics, conjunctivitis	Fi, Fr, Le, Ba, Dfh	[49-51]	Diaphoretic, anti-inflammatory, diuretic	Fl, Ba	[27]

Alliaceae								

*Allium cepa *L.	cipuddra	[60]	To treat flu, cough and headache, antidiarrhoeic, vermifugue	Bl	[49]	Antibacterial, vermifugue, hypotensive, diuretic, hypoglycaemic, eupeptic	Bl	[27]

*Allium sativum *L.	agliu	[55,60]	To treat insect bites, neuralgias, calluses, rheumatisms, hypotensive, tinea, scabies, tooth decay, cold, diarrhoea	Bl, Cl	[49]	Hypotensive, antibacterial, hypoglycaemic, lipid-lowering, anti-inflammatory	Bl	[27]

Apocynaceae								

*Nerium oleander *L.	leandru	[58]	Not Reported	----	----	Cardiotonic	Le	[28]

Asteraceae								

*Achillea millefolium *L.	millefogghio	[58,71]	Emmenagogue	Ap	[49]	Eupeptic, cholagogic, choleretic, antidyspeptic	Fl, Ap	[27]

*Artemisia absinthium *L.	erba janca	[26,56,58,62,65,71]	Not Reported	----	----	Eupeptic, cholagogic, antidyspeptic	Fh, Le	[27]

*Centaurea benedicta *(L.) L.	centarva	[56,62,65,68]	Not Reported	----	----	Eupeptic	Fh, Le	[28]

*Centaurea centaurium *L.	centu gruppa	[11]	Not Reported	----	----	Antioxidant	Ro	[29]

*Matricaria chamomilla *L.	gamumilla	[26]	Digestive, sedative, antispasmodic, antitussive	Fh	[49,50]	Anti-inflammatory, antibacterial, antifungal	Fh	[27]

Boraginaceae								

*Borago officinalis *L.	erva pignola	[60]	Diaphoretic, reddenings, burns, sunburns, tussis, rheumatisms, refreshing, diuretic	Le, Ap	[49,50]	Anti-inflammatory	Oi	[27]

*Heliotropium europaeum *L.	Not reported	[67]	Urinary inflammations	Ap	[49]	Antibacterial	Oi	[30]

Capparaceae								

*Capparis spinosa *L.	chiappara	[62]	Not Reported	----	----	Antiviral	Bl	[31]

Cactaceae								

*Opuntia ficus indica *(L.) Mill.**	ficuniano	[60]	Antispasmodic, antidiarrhoeic, diuretic, to treat bronchitis,	Fl, Fr	[49]	Anti-inflammatory	Cld	[32]

Cucurbitaceae								

*Ecballium elaterium *(L.) A. Rich.**	cucummeru	[57]	Antirheumatic	Fr	[51]	Cholagogic	Frj	[33]

Dryopteridaceae								

*Dryopteris filix-mas *(L.) Schott.	filici masculu	[58]	To treat sores provoked by severe burns	Le	[49]	Anti-helmintic, anti-amebic, antiparasitic, antiprotozoal	Ro	[34]

Euphorbiaceae								

*Ricinus communis *L.	ricinu	[26]	Not Reported	----	----	Laxative	Se	[28]

Fabaceae								

*Lupinus albus *L.	lupinu	[56,58,62,68]	To treat dermatitis in cattle	Ep	[50]	Gingival anti-inflammatory	Se	[35]

Fagaceae								

*Quercus *spp.	cerza	[55,58]	Intestinal astringent, detoxifying, cicatrizing	Dried bark of young stems and galls	[50]	Anti-inflammatory, antidiarrhoeic	Ba	[27]

Gentianaceae								

*Erythraea centaurium *(L.) Borkh.	brundulija	[11,60]	Not Reported	----	----	Eupeptic, antidyspeptic	Le, Fl	[27]

*Gentiana lutea *L.	genziana	[55]	Not Reported	----	----	Antidyspeptic, eupeptic	Ro	[27]

Juglandaceae								

*Juglans regia *L.*	nuci	[68]	Vermifuge, anti-diarrhoeic, stomachic, to remove calluses, against excessive feet perspiration	Hu, Le, Fr, Fle	[49-51]	Anti-inflammatory	Le	[27]

Hyacinthaceae								

*Urginea maritima *(L.) Baker	cipuddazza	[56,62]	Not Reported	----	----	Cardiotonic	Bl	[28]

Lamiaceae								

*Ajuga chamaepitys *Guss.	campezio	[72]	Not Reported	----	----	anabolic, analgesic, anti-arthritic, antibacterial, antiestrogenic, antifungal, anti-inflammatory, anti-hypertensive, antileukemic, antimalarial antimycobacterial, antioxidant, antipyretic, cardiotonic, cytotoxic, hypoglycemic, vasorelaxing activity°°	Ep	[36]

*Ballota nigra *L.	marrobio nero	[63]	Not Reported	----	----	Antioxidant	Le	[44]

*Calamintha nepeta *(L.) Savi	nipitella	[64,69,70]	To cure insect and snake bites, cicatrizing	Fle, Fh	[49,50]	Antibacterial	Eoi	[45]

*Hyssopus officinalis *L.	issopu	[65]	Not Reported	----	----	Emmenagogue	Ro	[27]

*Rosmarinus officinalis *L.	rosimarinu	[64,70,71]	To ripen abscesses quickly, carminative, digestive, to speed up the recovery of sores and wounds	Fl, Le, Br	[49]	Eupeptic, antidyspeptic, emmenagogue, anti-inflammatory	Le, Fh, Eoi	[27]

*Salvia officinalis *L.	sarvia	[64,70,71]	Digestive, antiasthmatic, to speed up the recovery of sores and wounds, to treat tussis, to cure aphtas and stomatitis, to treat swollen testicles and related pains	Le, Fl, Dle	[49,50]	Antioxidant, anti-inflammatory	Le, Ap	[27]

*Teucrium chamaedrys *L.	cametriu	[11,26,55,56,62,65]	Not Reported	----	----	Poisonous	----	[37]

Lythraceae								

*Punica granatum *L.	granato	[58]	Haemostatic, vermifuge	Frb, Rob	[51]	Emmenagogue	Rob	[27]

Myrtaceae								

*Eucalyptus *spp.	calipsi	[58,68]	Antiseptic of the respiratory tract	Le	[50]	Antibacterial, anti-inflammatory, spasmolytic, expectorant	Le	[27]

Oleaceae								

*Olea europaea *L.	alivu	[62,68]	Cholagogic, hypotensive, astringent, suppurative, to treat small burns, tooth ache	Le, Rfr, Ba, Oi	[49-51]	Hypotensive, diuretic, spasmolytic, antipyretic	Le	[27]

Papaveraceae								

*Chelidonium majus *L.	cucumaju	[56]	To treat warts, calluses, gastric pains	La, Le	[49]	Cholagogic, choleretic, hypotensive, antibacterial, antifungal, antiviral, anti-inflammatory, antidyspeptic	Ap	[27]

*Fumaria officinalis *L.	fumaria	[11]	Not Reported	----	----	Cholagogic, choleretic	Ap	[27]

Piperaceae								

*Piper nigrum *L.	pipi nivuro	[60,63,64]	Not Reported	----	----	Antioxidant, anti-inflammatory, anti-diarrhoeal, eupeptic	Se	[46]

Poaceae								

*Arundo donax *L.	canna	[62]	Haemostatic, cicatrising, to treat throat inflammations and bronchitis	Sa, Rh	[49,50]	Hypotensive, spasmolytic	Rh	[38]

*Cynodon dactylon *(L.) Pers.	acropastu, addisa, gramigna	[11,54]	Diuretic, to alleviate rheumatic pains, inflammations of the digestive and urogenital system	Ap, Rh, Se	[49-51]	Diuretic, anti-inflammatory	Ro	[27]

Rosaceae								

*Prunus spinosa *L.	cucumele	[24,69,72,73]	Not Reported	----	----	Anti-inflammatory	Fr	[27]

Rutaceae								

*Citrus bergamia *Risso	bergamotto	[64]	Cicatrizing and antiseptic for wounds and chilblains, to cure anomalous vaginal secretions, as a contraceptive	Eoi	[49]	Antimicrobial	Ba	[47]

*Citrus limonum *Risso	limuni	[11,56,65,74]	Stomachic, to treat cough, slimming agent, chilblains, migraine (after drunkenness), toothache, rheumatisms, oral hollow diseases	Frj, Fr,	[49]	Anti-inflammatory	Frj	[39]

*Ruta *spp.	ruta	[64,70]	Anti-helmintic, to treat gastritis, abscesses, rheumatic pains, headache, intestinal inflammations and eye reddening	Ap, Le	[49,50]	Antibacterial	Le	[48]

Salicaceae								

*Salix *spp.	salici	[58]	Against fever and rheumatic pains	Ba	[50]	Antipyretic, anti-inflammatory, analgesic	Ba	[27]

Smilacaceae								

*Smilax aspera *L.	strazza buttuni	[11]	Not Reported	----	----	Adaptogen	Ro	[27]

Solanaceae								

*Solanum dulcamara *L.	durcamara	[11]	Not Reported	----	----	To treat dermatitis	St	[27]

*Capsicum annuum *L.	pipariaddru	[56,60,62,75]	To rise blood flow to superficial tissues	Fr	[49]	Antidyspeptic, anti-inflammatory	Fr	[27]

*Capsicum annuum *L. Var. *acuminatum *Fing.	pipi	[55,57,60,68,76]	Revulsive	Fr	[49]	Antioxidant	Fr	[40]

Tamaricaceae								

*Tamarix *spp.	vruca	[24,65]	Not Reported	----	----	Antioxidant, antibacterial	Fl, Le	[41]

Urticaceae								

*Parietaria officinalis *L.	erba 'i muru	[56,62]	Diuretic, depurative, cholagogue, to treat bruises, haematoma, kidney stones, abscesses, skin inflammations, viper bite	Le, Ro, Ap, Ep	[49-51]	Diuretic, uricosuric	Ap	[42]

*Urtica dioica *L.	urdica	[62]	Antirheumatic, hepatoprotective, to treat haemorrhoids, renal troubles	Ap, Ro, Le, To	[49,50]	Diuretic, anti-inflammatory	Ap, Ro	[27]

*Urtica urens *L.	urdica piccola	[63]	Not Reported	----	----	Diuretic, anti-inflammatory	Ap, Ro	[27]

Valerianaceae								

*Valeriana officinalis *L.	malariana	[65]	Not Reported	----	----	Sedative	Ro	[27]

Verbenaceae								

*Verbena officinalis *L.*	erba di la crucivia	[56,57,62]	Not Reported	----	----	Anti-inflammatory, analgesic	Le	[43]

* Plant used like magic remedy too (see text); ** plants used like magic remedy only (see text); Used parts of plant: Ap aerial part; Ba bark; Bl bulb; Br branches; Bu bud; Cl cloves; Cld cladodes; Dle dry leaves; Dfh dry flower heads; Eoi essential oil; Ep entire plant; Fh flower heads; Fi feminine inflorescences; Fl flowers; Fle fresh leaves; Fr fruit; Frb fruit bark; Frj fruit juice; Hu husk; La latex; Le leaves; Oi oil; Rfr ripe fruit; Rh rhizome; Ro root; Rob root bark; Sa sap; Se seeds; St stem; To tops;

° Current use drawn from recent fieldworks

°° The properties relating to certain species of the genus Ajuga

The family names of the plants recorded in this work follow the Angiosperm Phylogeny Group guidelines [52].

## Results

### Empirical remedies

The empirical remedies that were used by the Calabrian people, as evidenced by the sources consulted in this work, were used both prophylactically and therapeutically, and were based on drinks, objects, animals, plants and other sources; some of these elements were created *ex novo*, while others were inherited from the "official" medicine of 1th-3th century AD [Tables 3 and 4].

**Table 3:** Medical use of plants to heal malaria in Calabria, mentioned by historical sources used for the research.

Plants Family/Scientific Name	Pliny	Dioscorides	Galen	Serenus Sammonicus
Adoxaceae				

*Sambucus nigra *L.	To cure dropsy	To cure dropsy	To cure spleenomegaly	To cure dropsy
	(*Naturalis Historia *XXIV, 52)	(*De Materia Medica *IV, 172)	(*Galeni Opera Omnia *XIII, 244)	(*Liber Medicinalis *XXVI, 498)
	[13]	[16]	[21]	[22]

Alliaceae				

*Allium cepa *L.		To cure dropsy		
		(*De Materia Medica *II, 181)		
		[16]		

*Allium sativum L.*	To cure quartain fevers			To cure quartain fevers
	(*Naturalis Historia *XX, 23)			(*Liber Medicinalis *XLIX, 899)
	[15]			[22]

Apocynaceae				

*Nerium oleander L.*				

Asteraceae				

*Achillea millefolium L.*				

*Artemisia absinthium L.*	To cure hepato-spleenomegaly	To cure dropsy and spleenomegaly	To cure spleenomegaly	To cure quartain fevers
	(*Naturalis Historia *XXVII, 28)	(*De Materia Medica *III, 23)	(*Galeni Opera Omnia *XIII, 240)	(*Liber Medicinalis *XLIX, 903)
	[15]	[16]	[21]	[22]

*Centaurea benedicta *(L.) L.				

*Centaurea centaurium *L.		To cure fevers	To cure fevers	
		(*De Materia Medica *III, 6)	(*Galeni Opera Omnia *XII, 19)	
		[16]	[20]	

*Matricaria chamomilla *L.		To cure fevers		
		(*De Materia Medica *III, 144)		
		[16]		

*Boraginaceae*				

*Borago officinalis *L.				

*Heliotropium europaeum *L.	To cure quartain fevers	To cure tertian and quartain fevers		
	(*Naturalis Historia *XX, 29)	(*De Materia Medica *IV, 190)		
	[15]	[16]		

Capparaceae				

Capparis spinosa L.	To cure spleenomegaly	To cure spleenomegaly	To purge; To cure spleenomegaly and dropsy	
	(*Naturalis Historia *XX, 59)	(*De Materia Medica *II, 204)	(*Galeni Opera Omnia *XII, 9)	
	[15]	[16]	[20]	

Cactaceae				

*Opuntia ficus indica *(L.) Mill.**				

Cucurbitaceae				

*Ecballium elaterium *(L.) A. Rich.**		To cure dropsy	To cure jaundice	
		(*De Materia Medica *IV, 154)	(*Galeni Opera Omnia *XII, 122)	
		[16]	[20]	

Dryopteridaceae				

*Dryopteris filix-mas *(L.) Schott.		To cure spleenomegaly		To cure dropsy
		(*De Materia Medica *IV, 158)		(*Liber Medicinalis *XVI, 511)
		[16]		[22]

Euphorbiaceae				

*Ricinus communis *L.		To purge; To cure dropsy	To purge	
		(*De Materia Medica *IV, 141)	(*Galeni Opera Omnia *XII, 26)	
		[16]	[20]	

Fabaceae				

*Lupinus albus *L.	To cure spleenomegaly	To cure spleenomegaly		
	*(Naturalis Historia *XXII, 74*)*	(*De Materia Medica *II, 132)		
	[15]	[16]		

Fagaceae				

*Quercus *spp.		To expel urine		
		(*De Materia Medica *I, 143)		
		[16]		

Gentianaceae				

*Erythraea centaurium *(L.) Borkh.		To cure dropsy	To cure spleenomegaly	
		(*De Materia Medica *III, 7)	(*Galeni Opera Omnia *XII, 20)	
		[16]	[20]	

*Gentiana lutea *L.		To cure hepatomegaly		
		(*De Materia Medica *III, 3)		
		[16]		

Juglandaceae				

*Juglans regia *L.*				

Hyacinthaceae				

*Urginea maritima *(L.) Baker	To cure dropsy	To cure dropsy and jaundice	To cure hepato-spleenomegaly; To expel urine	
	(*Naturalis Historia *XX, 100)	(*De Materia Medica *II, 102)	(*Galeni Opera Omnia *XI, 746, 749)	
	[15]	[16]	[18]	

Lamiaceae				

*Ajuga chamaepitys *Guss.	To cure dropsy	To cure jaundice	To expel urine; To cure spleenomegaly	
	(*Naturalis Historia *XXIV, 30)	(*De Materia Medica *III, 175)	(*Galeni Opera Omnia XII *155; XIII, 240)	
	[13]	[16]	[20,21]	

*Ballota nigra *L.			To cure spleenomegaly	To cure hepato-splenomegaly
			(*Galeni Opera Omnia *XII, 108)	(*Liber Medicinalis *XXII, 417)
			[20]	[22]

*Calamintha nepeta *(L.) Savi		To cure jaundice	To cure dropsy	To cure spleenomegaly and dropsy
		(*De Materia Medica *III, 28)	(*Galeni Opera Omnia *XIII, 264)	(*Liber Medicinalis *XXII, 419; XVI, 504)
		[16]	[21]	[22]

*Hyssopus officinalis *L.	To cure spleenomegaly	To cure dropsy and spleenomegaly	To cure dropsy	
	(*Naturalis Historia *XXVI, 48)	(*De Materia Medica *III, 28)	(*Galeni Opera Omnia *XIII, 263)	
	[13]	[16]	[21]	

*Rosmarinus officinalis *L.	To cure hepato-splenomegaly	To cure jaundice	To cure jaundice	To cure hepato-splenomegaly
	(*Naturalis Historia *XXIV, 59)	(*De Materia Medica *III, 89)	(*Galeni Opera Omnia *XII, 60)	(*Liber Medicinalis *XXII, 408)
	[13]	[16]	[20]	[22]

*Salvia officinalis *L.		To expel urine		To cure hepatomegaly
		(*De Materia Medica *III, 35)		(*Liber Medicinalis *XXI, 381)
		[16]		[22]

*Teucrium chamaedrys *L.	To cure spleenomegaly and dropsy	To cure spleenomegaly	To cure spleen; To expel urine	
	(*Naturalis Historia *XXIV, 131)	(*De Materia Medica *III, 102)	(*Galeni Opera Omnia *XII, 153)	
	[13]	[16]	[20]	

Lythraceae				

*Punica granatum *L.				

Myrtaceae				

*Eucalyptus *spp.				

Oleaceae				

*Olea europaea *L.		To expel urine		
		(*De Materia Medica *I, 141)		
		[16]		

Papaveraceae				

*Chelidonium majus *L.		To cure jaundice	To cure fevers	
		(*De Materia Medica *II, 211)	(*Galeni Opera Omnia *XII, 156)	
		[16]	[20]	

*Fumaria officinalis *L.		To expel urine	To expel urine	
		(*De Materia Medica *IV, 108)	(*Galeni Opera Omnia *XII, 8)	
		[16]	[20]	

Piperaceae				

*Piper nigrum *L.		To cure periodical fevers	To cure quartain fevers	To cure hepatomegaly
		(*De Materia Medica *II, 158)	(*Galeni Opera Omnia *XIV, 524)	(*Liber Medicinalis *XXI, 384)
		[16]	[19]	[22]

Poaceae				

*Arundo donax *L.	To cure dropsy			
	(*Naturalis Historia *XXIV, 50)			
	[13]			

*Cynodon dactylon *(L.) Pers.			To expel urine	
			(*Galeni Opera Omnia *XI, 810)	
			[18]	

Rosaceae				

*Prunus spinosa *L.				

Rutaceae				

*Citrus bergamia *Risso				

*Citrus limonum *Risso				

*Ruta *spp.		To cure dropsy	To expel urine; to cure dropsy	
		(*De Materia Medica *III, 45)	(*Galeni Opera Omnia *XII, 101; XIII, 257)	
		[16]	[20,21]	

Salicaceae				

*Salix *spp.				

Smilacaceae				

*Smilax aspera *L.				

Solanaceae				

*Solanum dulcamara *L.			To expel urine	
			(*Galeni Opera Omnia *XII, 145)	
			[20]	

*Capsicum annuum *L.				

*Capsicum annuum *L. Var. *acuminatum *Fing.				

Tamaricaceae				

*Tamarix *spp.	To cure spleenomegaly	To cure spleenomegaly	To cure spleenomegaly	To cure spleenomegaly
	(*Naturalis Historia *XXIV, 61)	(*De Materia Medica *I, 116)	(*Galeni Opera Omnia *XII, 80)	(*Liber Medicinalis *XXII, 408)
	[15]	[16]	[20]	[22]

Urticaceae				

*Parietaria officinalis *L.				

*Urtica dioica *L., *Urtica urens *L.	To cure spleenomegaly; to expel urine	To cure spleenomegaly		
	(*Naturalis Historia *XXII, 15)	(*De Materia Medica *IV, 102)		
	[15]	[16]		

Valerianaceae				

*Valeriana officinalis *L.		To cure jaundice	To expel urine	
		(*De Materia Medica *I, 6)	(*Galeni Opera Omnia *XII, 85)	
		[16]	[20]	

Verbenaceae				

*Verbena officinalis *L.	To cure fevers	To cure tertian and quartain fevers		
	(*Naturalis Historia *XXV, 59)	(*De Materia Medica *IV, 61)		
	[13]	[16]		

**Table 4:** Medical use of animals to heal malaria in Calabria, mentioned by historical sources used for the research.

Animals	Pliny	Dioscorides	Galen	Serenus Sammonicus
*Cimex lecturalius*	To cure quartain fevers	To cure quartain fevers		To cure tertian fevers
	(*Naturalis Historia *XXIX, 17)	(*De Materia Medica *II, 36)		(*Liber Medicinalis *XLIX, 921)
	[14]	[16]		[22]

*Homo sapiens*		To cure dropsy		
		(*De Materia Medica *II, 99)		
		[16]		

*Lumbricus terrestris*		To cure tertian fevers		
		(*De Materia Medica *II, 99)		
		[16]		

*Spider*	To cure quartain fevers	To cure quartain fevers		
	(*Naturalis Historia *XXX, 30)	(*De Materia Medica *II, 48)		
	[14]	[16]		

*Vipera aspis*	To cure fevers			
	(*Naturalis Historia *XXX, 30)			
	[14]			

*Capra hircus*		To cure hepatomegaly	To cure spleenomegaly and dropsy	
		(*De Materia Medica *II, 98)	(*Galeni Opera Omnia *XII, 297; XIII, 263)	
		[16]	[20,21]	

*Erinaceus europaeus*	To cure dropsy	To cure dropsy		
	(*Naturalis Historia *XXX, 30)	(*De Materia Medica *II, 2)		
	[14]	[16]		

*Cantharis vescicatoria*	To cure dropsy		To expel urine	
	(*Naturalis Historia *XXIX, 96)		(*Galeni Opera Omnia *XII, 363)	
	[14]		[20]	

*Snake*	To cure quartain fevers			
	(*Naturalis Historia *XXX, 30)			
	[14]			

*Bos taurus*			To cure dropsy	
			(*Galeni Opera Omnia *XIII, 263)	
			[21]	

*Empirical prophylactic remedies*. One of the most commonly used prophylactic empirical remedies was bleeding (which was already described by Galen that affirmed "*Saluberrimum igitur, ut praediximus, est in febribus venam incidere" *(during the fever, as mentioned, it is very useful to incise a vein) (*De Methodo Medendi *XI, 15) [17]. Bleeding was performed preventively by "barbieri" (barbers) and "magare" (witches) during the month of March [53]. During the same period (when the cure was called "marziale"), Calabrian people drank different types of decoctions, such as those made with "durcamara" (*Solanum dulcamara *L.), "acropastu" (*Cynodon dactylon *(L.) Pers.), "strazza buttuni" (*Smilax aspera *L.) and "fumaria" (*Fumaria officinalis *L.) [11,54]. To prevent contagion of the disease during the night in the summer months, people slept for few hours and near a fire [54-56]. Moreover, they drank strong spirits or wine. In particular, they were advised to drink half a litre of wine on an empty stomach [57,58], eat garlic (*Allium sativum *L.) [59], smoke and chew tobacco and swallow the spittle [56,60], while always maintaining the pipe in the mouth [54-56]. On awaking, it was recommended to eat a macerate of raw garlic in vinegar [54]. Finally, people living on the coast used to spread olive oil mixed with absinth on their bodies, according to Dioscorides who affirmed "*Itemque ex oleo perunctum, culices abigere, ne corpus tangant*" (*Rubbed on with oil it forbids the mosquitos to touch the body*) *De Materia Medica*, III, 23) [16,61] and according to Pliny "culices ex oleo perunctis abigit" (who use this oil keep mosquitos away), *Naturalis Historia *XXVII, 28) [13].

*Empirical therapeutical remedies*. Fasting and purging were recommended for the treatment of malaria-associated fevers. Fasting was thought to appease fever, while purging was thought to remove the malaria-causing parasite from the affected organism. In general, purging was achieved via the administration of ricinus seeds (*Ricinus communis *L.) and by using the root of "savucu" (*Sambucus nigra *L.) [26]. Fasting and purging were inherited from Galenic medicine. Galen wrote the following about fasting: "*(in tertiariis) ... neque quotidie cibum dare oportet, sed alternis diebus abunde fuerit*" (with tertian fevers ... food must not be offered every day, but on alternate days) (*Ad Glauconem de medendi methodo *I, 11) [18]; and about purging: "*ac vacuatio quidem excrementorum omni febri est utilissima*" (during the fever, no doubt, it is very useful to defecate) (*Methodo medendi *IX, 10) [17]. Other treatments aimed at purging and restoring the affected subjects were also used. These included the decoction of "gamumilla" (*Matricaria chamomilla *L), "ordica" (*Urtica dioica *L., *Urtica urens *L.), and the decoction of the root of "alivu" (*Olea europaea *L.) or of the rhizome of "canna" (*Arundo donax *L.) [26,62,63]. Several empirical therapeutic remedies against malaria-associated fevers were loathsome. These included the ingestion of the subject's own urine, that of young virgin or that of a healthy woman (in particular, the affected subject was advised to drink 100 g of the urine of a non-affected woman early in the morning) [55-57,64], the consumption of various animals (or parts of animals), such as earthworms (*Lumbricus terrestris*) which were previously placed in the oven and pulverized, or two or three bedbugs (*Cimex lecturalius*) within a Host [56,58,64]. Moreover, patients were encouraged to eat pills of "*pappici*" (cobweb) [26,56,63-66], the head of a viper (*Vipera aspis*), fried and mixed with absinth [55], goat (*Capra hircus*) dung within a Host, eaten from morning until midday [26,57], one spoonful of coffee per hour [57] and pills of soot [67,68]. Alternatively, to cure hepato-spleenomegaly were used hedgehog (*Erinaceus europaeus*) or ox (*Bos taurus*) gall and goat (*Capra hircus*) dung [55]. As three "Cantarelle" (*Cantharis vescicatoria*) minced in water were used as a diuretic against dropsy [56].

Calabrian people believed without any doubt in the remedies described above; however, they also used numerous plants to cure malaria. Some of these plants are still currently used in Calabrian folk medicine to cure various diseases [Table 1]. Many decoctions or infusions of various herbaceous species were used to cure malaria-associated fevers. These herbaceous plants included "issopu" (*Hyssopus officinalis *L.), "valariana" (*Valeriana officinalis *L.), "filici masculu" (*Dryopteris filix-mas *(L.) Schott.), "lupinu" (*Lupinus albus *L.), "cametriu" (*Teucrium chamaedrys *L.), "brundulija" (*Erythraea centaurium *(L.) Borkh.), "centu gruppa" (*Centaurea centaurium *L.), "centarva" (*Centaurea benedicta *L.), "sarvia" (*Salvia officinalis *L.), "rosamarinu" (*Rosmarinus officunalis *L.), "nepitella" (*Calamintha nepeta *(L.) Savi), "ruta" (*Ruta *spp.), "erva janca" (*Artemisia absinthium *L.), "cipuddra" (*Allium cepa *L.), "agliu" (*Allium sativum *L.), "millefoglio" (*Achillea millefolium *L.), "erva pignola" (*Borago officinalis *L.), "marrobio nero" (*Ballota nigra *L.), "campezio" (*Ajuga chamaepitys *Guss.), "elitropia" (*Heliotropium europaeum *L.) (which was ingested with white wine), "genziana" (*Gentiana lutea *L.) and "erba i la crucivia" (*Verbena officinalis *L.) [11,26,55-58,60,62-65,67-72]. Some ligneous species must be added to this list, particularly the following plants: the aerial parts of "vruca" (*Tamarix *spp.), and "leandru" (*Nerium oleander *L.); the leaves of "alivu" (*Olea europaea *L.) and "calipsi" (*Eucalyptus *spp.); the roots of "granato" (*Punica granatum *L.), "cucumele" (*Prunus spinosa *L.), "savucu" (*Sambucus nigra *L.), and "cerza" (*Quercus *spp.). The bark of "cucumele", "cerza" and "salici" (*Salix *spp.) was also used [24,26,55,58,62,65,68,69,72,73].

Other remedies were similarly efficacious; these included the mesocarp of "nuci" (*Juglans regia *L.) chopped finely and mixed with wine, "bergamotto" (*Citrus bergamia *Risso), "limuni" (*Citrus limonum *Risso, which was broken, boiled and maintained fresh overnight, then drunk at breakfast for three mornings), "pipi nivuru" (*Piper nigrum *L.), "pepe arsente" (*Capsicum annuum *L.) and ten bitter seeds of decorticated "lupinu" (*Lupinus albus *L.), taken in the morning [11,56,58,60,62-65,68,74,75].

Other than fever, the most evident symptoms of malaria are hepato-spleenomegaly and dropsy. We also found descriptions of several remedies for these symptoms. There were many cures for hepato-spleenomegaly: a decoction of the root of "chiappara" (*Capparis spinosa *L.), or of "acropistu" (*Urtica dioica *L., *Urtica urens *L.), the latter taken together with potassium nitrate in the morning; eating "cipuddra" (*Allium cepa *L.) or "pipi" (*Capsicum annuum *L. Var. *acuminatum *Fing.), the latter together with a strong wine. Finally, another remedy involved the use of "cucumaju" (*Chelidonium majus *L.) [55-57,60,63,68,76].

To treat dropsy, which was called "acqua 'ntà panza" (water in the stomach), Calabrian people used several diuretic remedies, such as "erba i muru" (*Parietaria officinalis *L.), "cipuddazza" (*Urginea maritima *(L.) Baker), and "cametriu" (*Teucrium chamaedrys *L.) [11,26,55,56,62,66].

### Magic remedies

Because of the presence of malaria in the daily lives of Calabrian people, this disease was considered a normal life trouble; however, its most dangerous and deadly forms were considered by Calabrian people as a condition of supernatural nature. Therefore, they resorted to magic remedies that were believed to "link" the disease. These included, in particular, wearing a "nuci trischéra o a tri guarri" (a three-valve walnut shell) (*Juglans regia *L.), a spider that was enclosed between two shells of a walnut or skin, skeleton and fangs of snake, the latter extracted when animal was still alive, as it was believed that the disease would then affect the walnut, the spider or the parts of the snake, and not the subjects who wore these amulets [65,77]. Furthermore, a live "carpurita" (*Pachyiulus communis*) was sewn into the clothes of the affected subject (without the patient realizing it) or a "paletta" (*Opuntia ficus-indica *(L.) Mill.) was placed near the fireplace. It was believed that when the animal died, or when the stem of the plant dried, the fever or the hepato-spleenomegaly would disappear [26,60]. In an analogy with the ancient belief in the therapeutic principle of "contact", to defeat spleenomegaly Calabrians were encouraged to place "erba i la crucivia" (*Verbena officinalis *L.) on the abdomen of the affected subject before sleeping, as it would absorb the "bad blood" [56,57,62]. Finally, every morning the affected subject had to urinate on "cucuzzielli acriesti maturi", the fruits of *Ecballium elaterium *(L.) A. Rich., to transfer the disease from the subject to the fruit [57].

### Religious remedies

Calabrian people alternated or combined both empirical and magical remedies and, very often, used prayers and acts of devotion, as diseases were believed to be associated to divine punishment. Thus, in Cosenza (Northern Calabria) the "Madonna della Febbre" was invoked with prayers, *ex voto *and pilgrimages [78]; in Castrovillari (province of Cosenza), the prayer to the "Madonna d'Itria" was as follows: "*Madonna mia 'i L'Itria, chi stai 'nganna a'sta jumara fammi passà 'sta freva 'i quartana c'u jurnu tuju non vugghiu mangià panu*" ("My Lady of Itria, close to the river, let the fever out and on your commemoration day I will not eat bread") [79].

## Discussion

The methodology based on the analysis of historical sources regarding Calabrian folk medicine remedies for the prophylaxis and treatment of malaria, if not compared with similar studies, can be considered a case study where the ordinary methodologies of ethno-medical-biological research are combined with the methodologies pertaining to historical-anthropological sciences. In addition, this is part of a debate regarding the association between ethnobotany and ethnopharmacology and other disciplines, to improve our understanding of the human usage of plants [80]. Moreover, this work complies with De Natale et al. [81], who created a database of the historical use of plants in the popular medicine of the Mediterranean basin. However, this study has revealed some interesting and heterogeneous features regarding Calabrian popular medicine practices used to prevent and treat malaria, some of which were inherited by the Calabrian people from the "expert medicine" of the past centuries.

The first type of practices that we have described were characterized by a rational approach. Indeed, the use of medicinal plants, 69% of which (36/52) is recognized by the current pharmacopoeia as having some pharmacological/biological properties, succeeded in assuaging temporarily the most evident sufferings associated with the disease (fever, hepato-spleenomegaly, asthenia and dropsy) as well as its complications, such as the proneness to bacterial infections, even if did not cure the malarial infection. 23% (12/52) of the plants which were used by Calabrian people to treat malaria, have pharmacological/biological properties which did not allow to relieve the symptoms of malaria; however, they did not damage the affected subjects. Finally, 8% (4/52) of these plants were characterized by some pharmacological/biological properties which could be harmful for a malarial subject; or these properties could even be poisonous both for the malarial and the healthy subject.

The second type of practices were linked to the magic tradition of Calabrian folk medicine which, like the traditions of all Southern Italian regions, is rich in myths, symbolism and fantastic representations [82]. Thus, malaria became a synonym of "malia", or, as Pasquarelli [83] affirmed, it became "an aspect of paludism". Malaria was thought to be a consequence of a malefic element that affected the behaviour and the life of an individual; therefore, only a magic cure could remedy the disease.

The third type of practices were characterized by a strong principle of ineluctability, which is currently present among the Calabrian society: the sick entrusted God with prayers or acts of devotion, with the conviction that only God would be able to provide recovery from the disease.

## Conclusions

The use of plants combined with other cures, such as the use of spiders, cantharis and leeches, represents prophylactic or therapeutic elements inherited from ancient medical science, some of which were still used to treat malaria in hospitals and in general by 19^th^-century physicians, before the introduction of quinine. This element is very interesting; while the empirical and magic remedies were not based on the symptomatology of the disease (they were rather "psychological and protective" elements [82]), the use of plants represented a real treatment, and served as a popular medicine base to treat various diseases.

In conclusion, the remedies described in this work allow us to establish the link between malaria and Calabrian people, so that Turner's statement that "the more widely or intensively a plant is used, the greater is its cultural significance" [84] can, in this case, be extended to malaria; the more folk remedies are used to cure malaria, the greater is the significance of its historical, medical and social meaning.

## Competing interests

The authors declare that they have no competing interests.

## Authors' contributions

GT conceived of the study, collected and analyzed the data, drafted the manuscript. AT and AP supervised the work at all its stages.
